# Establishing a new emergency department: effects on patient flow

**DOI:** 10.1186/cc12198

**Published:** 2013-03-19

**Authors:** V Rautava, T Valpas, M Nurmikari, A Palomäki

**Affiliations:** 1 Kanta-Häme Central Hospital, Hämeenlinna, Finland; 2 City of Hämeenlinna, Finland

## Introduction

Overcrowding in emergency departments (EDs) is a widely known problem. It causes problems and delays in the ED and has a negative impact on patient safety [1]. The aim of this study was to analyse whether a reform of emergency care can reduce patient flow into the ED.

## Methods

A substantial reform of emergency care took place in the province of Kanta-Häme in Southern Finland. Three separate out-of-hours services in primary healthcare (PHC) and one ED in the hospital were combined into one large ED in April 2007. Basic principles of the new ED were: the ED is only for those patients who are seriously ill or injured, and need immediate care; PHC (healthcare centres) take care of acute ordinary illnesses and nonserious injuries during office hours. To achieve these principles a regional five-scale triage system was planned and implemented. The information plan was established. Citizens were systematically informed about the principles of the new ED by mail, articles in the newspapers and interviews in the radio and television. The ED's Internet pages were planned and established. The number of patient visits (Hämeenlinna region) was analyzed 2 years before and after establishing the new ED.

## Results

**Figure 1:**
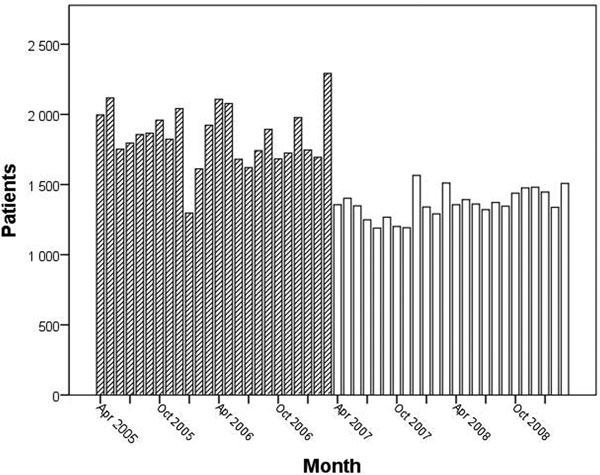
**GP patient visits before and after the reform of emergency services**.

During the 2-year period before the establishment of the new ED the mean number of GP patient visits was 1,845 ± 43/month. During the 2-year period after the reform the number was diminished to 1,364 ± 21/month. This change was not associated with the increase of the patient visits taken care of by specialists and hospital residents. See Figure 1.

## Conclusion

An extensive reform of the emergency services can notably reduce patient flow into the ED.
